# Monotonic Coding of Numerosity in Macaque Lateral Intraparietal Area

**DOI:** 10.1371/journal.pbio.0050208

**Published:** 2007-07-24

**Authors:** Jamie D Roitman, Elizabeth M Brannon, Michael L Platt

**Affiliations:** 1 Department of Neurobiology, Duke University, Durham, North Carolina, United States of America; 2 Department of Psychology and Neuroscience, Duke University, Durham, North Carolina, United States of America; 3 Center for Cognitive Neuroscience, Duke University, Durham, North Carolina, United States of America; Service Hospitalier Frederic Joliot, France

## Abstract

As any child knows, the first step in counting is summing up individual elements, yet the brain mechanisms responsible for this process remain obscure. Here we show, for the first time, that a population of neurons in the lateral intraparietal area of monkeys encodes the total number of elements within their classical receptive fields in a graded fashion, across a wide range of numerical values (2–32). Moreover, modulation of neuronal activity by visual quantity developed rapidly, within 100 ms of stimulus onset, and was independent of attention, reward expectations, or stimulus attributes such as size, density, or color. The responses of these neurons resemble the outputs of “accumulator neurons” postulated in computational models of number processing. Numerical accumulator neurons may provide inputs to neurons encoding specific cardinal values, such as “4,” that have been described in previous work. Our findings may explain the frequent association of visuospatial and numerical deficits following damage to parietal cortex in humans.

## Author Summary

As any child knows, to answer the question “how many,” one must start by adding up individual objects in a group. Extending beyond humans, this cognitive ability is shared by animals as diverse as birds and monkeys. Surprisingly, the exact brain mechanisms responsible for this process remain unknown. Damage to a brain area known as the parietal cortex disrupts basic mathematical skills, and functional imaging studies show that this area is activated when people perform basic computations. To understand how parietal cortex contributes to numerical behavior, we studied the activity of neurons in this area in monkeys while they looked at arrays of dots on a computer screen. We found that parietal neurons responded progressively as the total number of elements in the display was varied across a wide range of values (2–32). These neurons resemble “accumulator neurons” that have been suggested to serve as the first stage in counting. This information could be used by other neurons that respond best for a particular cardinal number, such as “4,” as has been reported in prior studies. Our findings support computer models that separate the processes of summing and numerical identification, and may also explain the fact that parietal cortex damage causes both numerical and spatial confusion.

## Abstract 3

Neurons in the lateral intraparietal area (LIP) in monkeys respond in a graded fashion to the number of items in a visual array during a delayed saccade task, suggesting that the neurons "sum up" individual elements to represent accumulated magnitude.

## Introduction

Humans and animals perform similarly in tasks that require representing the numerosity of a visual or auditory array, or making a given number of responses, suggesting that the underlying representation of quantity across species is similar [1–4]. It has been speculated that quantitative judgments are based on an analog magnitude representation of quantity akin to a “mental number line” that is either logarithmically compressed or linearly scaled with variance that increases proportionally with number [3,5]. Both lesion and functional imaging studies suggest that numerical processing is supported by neurons near the intraparietal sulcus in humans [6–10]. Moreover, in monkeys, neurons within the fundus of the intraparietal sulcus as well as in prefrontal cortex (PFC) respond selectively to a specific number of elements in a visual array [11–13]. Together, these findings implicate parietal cortex as a critical component of the neural circuitry responsible for encoding the mental number line. The fact that brain regions sensitive to number lie within the dorsal visual processing stream suggests that numerical quantity may be derived from the visuospatial representations [14–16] processed in this cortical pathway.

In monkeys, single neurons in posterior parietal cortex (PPC) encode intended locations for eye movements [17–19] and reaches [20,21], and reflect elapsed time when this information guides saccades [22]. Neurons in the lateral intraparietal area (LIP) reflect decisions based on random-dot motion stimuli [23], reward expectation [24], and the probability of a initiating a behavioral response to a particular location [24] or at a particular time [25]. All of these tasks rely on the accumulation of spatial, temporal, and reward information [26–28], thus it has been proposed that LIP neurons function as neural integrators that accumulate information relevant for guiding behavior [29,30].

**Figure 1:**
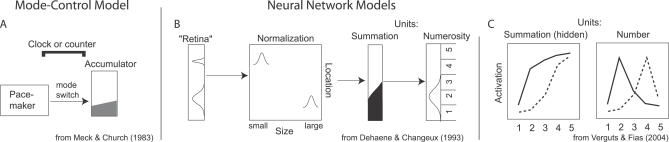
Models of Numerical Estimation Rely on an Accumulator Stage (A) In the mode-control model [31], an “accumulator” receives pulses from a pacemaker and stores an analog magnitude proportional to the quantity of stimuli enumerated. The accumulator value can represent elapsed time or quantity depending on the mode in which pulses are gated to it. (B) In the Dehaene and Changeux neural network model [32], “summation units” accumulate in parallel to the quantity of stimuli. This information is used by “numerosity” units, which represent the cardinal value of the set of stimuli. (C) The neural network model by Verguts and Fias [33] utilizes a hidden “summation” layer in which numerosity is represented in a monotonic fashion. Cardinal number is derived from summation units by “number” units.

Integration by neurons has also been proposed as a critical stage in computational models of numerical representation (Figure 1). The mode-control model by Meck and Church [31] proposes that number is represented by accumulating a fixed number of pulses from a pacemaker for each event or object enumerated (Figure 1A). Accumulation occurs as a *serial* process, with larger numbers represented by a greater accumulated magnitude over a longer interval. In a neural network model by Dehaene and Changeux [32], objects are represented on a retina as *n* areas of activation (Figure 1B). A normalization stage maps each object according to size and location, so that these attributes are not lost, but each object is represented by an equivalent amount of activation. “Summation” units integrate the activity on the normalization map in a *parallel* manner to extract stimulus quantity. “Numerosity” units then encode a specific cardinal value, such as 3 or 5, depending on the magnitude of the responses in the summation units. Similarly, a neural network model proposed by Verguts and Fias [33] utilizes hidden “summation” units that represent accumulated magnitude (Figure 1C). The outputs from these units are combined in an additive or subtractive manner to estimate cardinal value in “number” units.

In all these models, accumulator neurons are predicted to respond in a graded fashion with ordinal numerical quantity, in contrast with “numerosity” or “number” units that respond maximally for a particular cardinal value. Although prior studies have revealed evidence for a population of neurons selective for the cardinal number of elements in an array in both PFC and parietal cortex [11–13], evidence for putative numerical accumulator neurons remains elusive. A logical extension of the idea that neurons in LIP function as neural integrators is the hypothesis that these neurons accumulate numerical quantity. The inherent difficulties in dissociating sensitivity to number from sensitivity to space, salience, reward expectation, and motor preparation—all of which could potentially modulate neural responses—make this endeavor particularly challenging.

**Figure 2:**
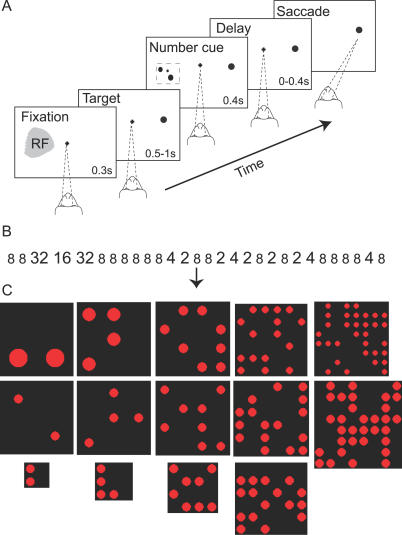
Implicit Numerical Discrimination Task (A) Sequence of trial events. The RF of the LIP neuron is first mapped using a delayed saccade task. Completion of each trial is followed by a fluid reward. (B) Sample trial sequence in which 8 is the standard numerosity and 2, 4, 16, and 32 are deviants. The standard is randomly presented on 50% of trials, and predicts a standard reward (0.1-ml fluid, indicated by smaller digit size). On the other trials, one of the deviant numerosities is shown, predicting a larger reward (0.15-ml fluid, larger digit size). Arrow identifies an example of numerosity that would be presented for an “8” trial. (C) Examples of dot arrays for the numerosities tested: 2, 4, 8, 16, and 32. Stimuli with the same average total number of pixels are in the first row; same radius and overall extent in the second row; and same radius and density in the third row.

To address this idea, we studied the activity of single neurons in parietal area LIP in monkeys performing an implicit numerical discrimination task (Figure 2A). This task permitted us to dissociate spatial sensitivity, attention, reward expectation, and motor preparation from neural coding of number. We controlled for spatial sensitivity by optimizing the position of the numerical stimulus so that it was placed in each neuron's classical spatial receptive field (RF). We dissociated salience from numerosity and reward expectation by presenting the same numerical value at different frequencies with different associated reward outcomes. On each trial, monkeys planned eye movement responses to a target located distal to the numerical stimulus, thus reducing any potential impact of motor planning on neural responses. In addition, we used a large range of numerosities (2–32), which facilitated discrimination of neurons representing accumulated quantity from neurons encoding cardinal numerical value. On each trial, the monkey viewed an array of 2, 4, 8, 16, or 32 elements located in the visual periphery for 400 ms. The numerosity of the array predicted the amount of reward the monkey would receive following a gaze shift to a response target in the opposite hemifield. On 50% of trials, a standard number was presented, predicting a standard reward size (0.10-ml fluid delivery, Figure 2B). On each of the remaining trials, a deviant number was shown, predicting a larger reward (0.15-ml fluid delivery). Standard and deviant numerosities were varied across blocks to dissociate reward-related salience and presentation frequency from visual quantity. Because complementary stimulus attributes often co-vary with numerosity, we also implemented stimulus controls to balance total number of pixels, element size, density, and, for completeness, color (Figure 2C).

We recorded the activity of single LIP neurons using standard techniques published previously [34] while monkeys performed the implicit numerical discrimination task. For each neuron, the monkey performed at least two blocks of this task so that each standard number was also tested as a deviant. This strategy permitted us to dissociate neural responses associated with numerosity from those driven by attention, which can be influenced by task variables like reward expectation or the frequency of presentation of particular numerical values. Notably, extensive training was not required since monkeys were only required to shift gaze to the response target; reward was delivered regardless of whether or not monkeys attended to the numerical cue (Figure 2B). In addition, we used up to 32 elements to study each neuron, a greater than 6-fold expansion over the numerosities used in prior electrophysiological studies of numerical representation. We hypothesized that the activity of LIP neurons would encode the quantity of visual elements placed within their classical RF in a graded manner, independent of low-level visual features, attention, or reward expectation.

## Results

Although monkeys were not required to process the numerical cue, we observed subtle but consistent effects of numerical deviance, and thus, expected reward size, on saccades. Response time (RT) between fixation offset and saccade initiation decreased as the numerical distance between standard and deviant increased (−5.57 ms for maximum difference of 30, confidence interval [CI]: −8.68 to −2.46, nested *F* = 12.33, *p* < 0.0001, Materials and Methods, Equation 1). The small magnitude of the difference in RT is not surprising, given the task was merely to shift gaze to a target that had been visible throughout the trial. Using similar tasks, other studies have also found small effects of reward size on saccade latency [35,36]. The subtle RT difference observed here suggests the monkeys attended to numerosity, despite no requirement to do so.

**Figure 3:**
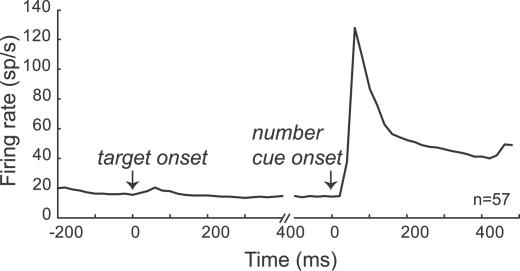
LIP Neurons Respond to the Onset of the Numerical Cue, Located within the RF, But Not to the Saccade Target, Located in the Opposite Hemifield For all 57 neurons studied, average firing rate was calculated in 20-ms bins. Trials are first aligned to the time that the saccade target appears (indicated by arrow). The break in the abscissa acknowledges the variable length of the delay before number cue onset. Following the appearance of the numerical cue in the RF (arrow), there is a large increase in LIP activity.

The monkeys' sole task was to shift gaze to a visible target following the disappearance of the fixation point (Figure 2A). Because LIP neurons are spatially selective [17–19], each neuron's response field was first mapped while monkeys made delayed saccades to visible targets (see Materials and Methods). To measure neural responses to the number of elements in a stimulus, we arranged the task geometry so that the entire numerical cue was presented in the neuron's RF and the saccade target was located in the opposite hemifield. For monkeys O and W, the average distances from the fixation point to the center of the RF, and thus the center of the numerical stimulus, were 15.2° and 17.5°, respectively. Previous work has shown that at these eccentricities, RF widths in LIP exceed the dimensions of the stimuli used here [37,38]. Figure 3 shows that onset of the saccade target did not evoke a neural response from the neurons studied. The neural population response remained low throughout the delay period (minimum, 500 ms) during which the target was present before the onset of the numerical cue, thus confirming the saccade response target was outside the classical RF. Presentation of the numerical cue itself elicited a large neural response, however, thus confirming its placement within the classical RF (Figure 3).

**Figure 4:**
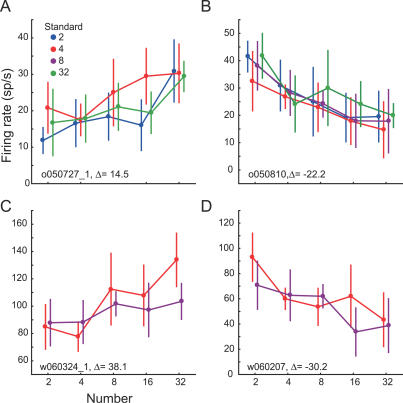
Single LIP Neurons Respond in a Graded Fashion to the Total Number of Elements in the Response Field Firing rate for each number (mean ± standard error [SE]) in the 400-ms epoch beginning 50 ms after stimulus onset, plotted for each standard block tested (line color). The change in firing rate (Δ) over the range of numerosities tested was estimated by linear regression with the logarithm of stimulus number (all *p* < 0.01; Materials and Methods, Equation 2). Numerical sensitivity did not depend on standard block (ANOVA, number × standard: [A] *p* = 0.59, [B] *p* = 0.99, [C] *p* = 0.10, and [D] *p* = 0.17). Neurons in (A) and (C) increased firing with larger numerosities, whereas those in (B) and (D) had larger responses for smaller numerosities.

Neuronal responses to the cue showed graded modulation by the numerosity of the stimulus. Figure 4 shows four examples of single LIP neurons with activity that either increased (Figure 4A and 4C) or decreased (Figure 4B and 4D) with increasing number of elements during stimulus presentation. In each example, firing rate depended on number (analysis of variance [ANOVA], *p* < 0.05), but not standard value (indicated by line color, ANOVA, *p* > 0.05). In the studied population, 35 neurons (61%) were sensitive to visual quantity (ANOVA, *p* < 0.05), and for the vast majority, (34/35, 97%), activity did not depend on which number served as standard or deviant (ANOVA, number × standard interaction, *p* > 0.05), thus effectively ruling out reward expectation or stimulus salience as factors modulating response to the numerical cue. For these examples, the modulation of firing rate over the range of numerosities was quantified using linear regression (Materials and Methods, Equation 2). We observed neurons with both increasing and decreasing numerical response profiles in both monkeys.

The majority of neurons modulated by number preferred either the smallest or largest numerosity tested. In previous studies [11–13], ANOVA was used to determine whether or not neurons were modulated by numerosity, with each neuron's “preferred” numerosity assigned as that which elicited the largest response. ANOVA thus provides a model-free means for assessing preferred numerosity. In our study, the majority of the neurons (30/35) found to be modulated by numerosity using ANOVA preferred either the largest or smallest value tested (Table 1).

**Table 1:**
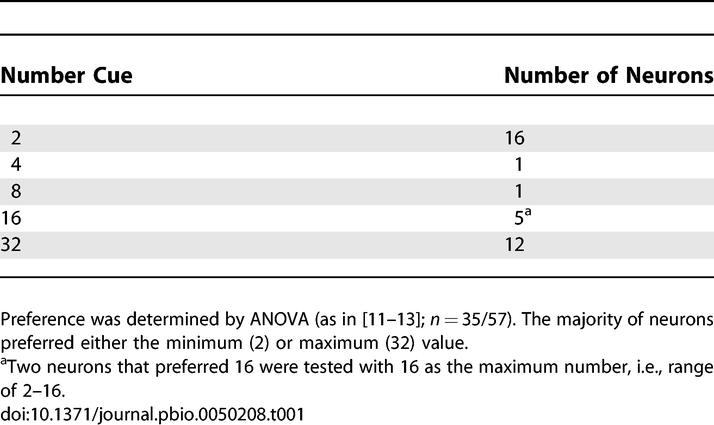
Preferred Numerosities for Neurons Found to Be Significantly Modulated by Number

Because we hypothesized that neural activity in LIP would encode the total number of elements within the RF in a roughly monotonic fashion, rather than encode a specific numerical value, we next used linear regression to quantify the relationship between firing rate and numerosity for all 57 neurons (Materials and Methods, Equation 2). Fourteen neurons had a significant positive relationship (as in Figure 4A and 4C), whereas 17 showed a significant negative relationship between numerosity and neural response (as in Figure 4B and 4D). An important question is whether the relationship between neuronal response and numerosity is scaled linearly or logarithmically. For 28 of these 31 neurons, firing rate varied significantly with both raw number and its logarithmic transform (Materials and Methods, Equation 2). For neurons with an increasing relationship between firing rate and number, the average correlation coefficients were 0.86 ± 0.23 (mean ± standard deviation) and 0.82 ± 0.18 for linear and logarithmic-scaled number, respectively. For neurons with a decreasing relationship between firing rate and number, the average correlation coefficients were −0.73 ± 0.13 for a linearly scaled number and −0.82 ± 0.11 for its logarithmic transform. Across these two groups of neurons, we compared how well the average population responses were fit when scaled linearly or logarithmically. Neurons with a negative relationship between firing rate and number were slightly better fit using the logarithmic transform of number (*F* = 3.54, *p* < 0.05), whereas there was no difference between firing rate and number for neurons with a positive relationship (*F* = 1.69, *p* = 0.19).

Thus, LIP neurons responded in a graded fashion to the total number of elements within their response fields. Our data also suggest that one subpopulation of LIP neurons was better described as systematically responding to number on a logarithmic scale. Therefore, we used the logarithmic transform of number in the remaining analyses; however, all analyses were repeated with raw numbers, and the same overall results were obtained.

**Figure 5:**
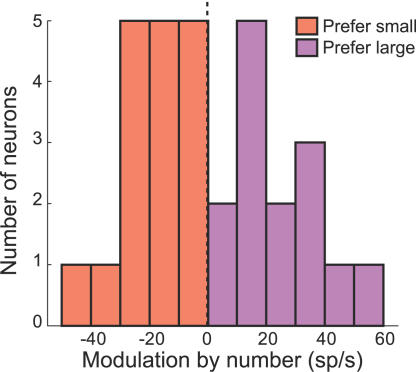
Sensitivity of the LIP Neuronal Population to the Total Number of Elements within the Response Field For each neuron, the Δ firing rate across range of numerosities was estimated by linear regression with the logarithmic transform of number (Materials and Methods, Equation 2). Red: neurons with significant negative slope (prefer small numerosity); purple: neurons with significant positive slope (prefer large numerosity).

The degree of response modulation by number, measured as the change in firing rate over the range of numerosities presented, for all numerosity-related neurons in the population, is shown in Figure 5 (Materials and Methods, Equation 2, *p* < 0.05). There was a strong correspondence between ANOVA and regression tests for numerical modulation: 28 neurons were found to have significant modulation by numerosity when tested by both ANOVA and regression, including the five cases in which the largest response was to an intermediate value. The proportion of neurons that preferred small or large values was not notably different in the two monkeys tested. Of the 31 neurons recorded from monkey O, 11 preferred small numerosities and eight preferred large, whereas of the 26 neurons recorded from monkey W, six preferred small numerosities and six preferred large. Thus, the majority of numerosity-sensitive neurons in our population were not tuned to cardinal numerical values, but instead represented numerosity in a roughly monotonic fashion—in stark contrast with neurons sensitive to cardinal numerical value reported in the ventral intraparietal area (VIP) and PFC.

**Figure 6:**
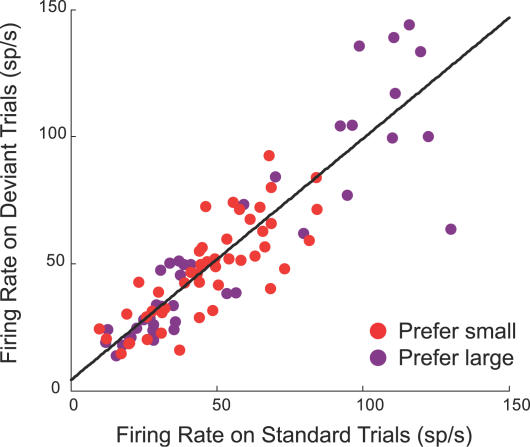
Numerical Sensitivity in LIP Does Not Reflect Attention or Reward Expectations For 31 neurons significantly modulated by number, the average response was compared for sets of trials in which a particular number was presented as both standard and deviant in the same recording session. The line shows the best fit to the data with an intercept of 5.5 sp/s and a slope of 0.92.

Modulation of neuronal activity by numerosity did not depend on whether the numerical cue was presented as the standard or as one of the deviant values. For the neurons with significant modulation by numerosity (Figure 5), we compared responses on trials in which the same numerical value was presented as both standard and deviant. Because neurons were typically tested in approximately three standard blocks, the comparison could not be made for all numerosities for every neuron. Figure 6 shows the average firing rate for all cases in which the same numerosity was tested as standard and deviant for a given neuron. The average firing rate did not systematically differ between standard and deviant trials. Nested regression was used to test whether there was modulation of firing rate due to the presentation of a given numerosity as standard or deviant in addition to the effect of numerosity. Across all neurons that preferred small numerosities, neural responses were modulated by numerosity, but not by assignment as standard or deviant (number: −20.31 spikes/s [sp/s], CI: −23.10 to −17.53, *F* = 102.9, *p* < 0.0001; standard: −0.06 sp/s, CI: −1.91 to 1.80, nested *F* = 0.003, *p* = 0.95; Materials and Methods, Equation 3). Similarly, neurons that preferred large numerosities showed no effect of presentation as standard or deviant on firing rate in addition to the effect on numerosity (number: 23.07 sp/s, CI: 18.0 to 28.2, *F* = 30, *p* < 0.0001; standard: −0.45 sp/s, CI: −3.76 to 2.87, nested *F* = 0.70, *p* = 0.79; Materials and Methods, Equation 3). Because there were no differences between neural responses on standard and deviant trials, all trials are included in subsequent analyses.

**Figure 7:**
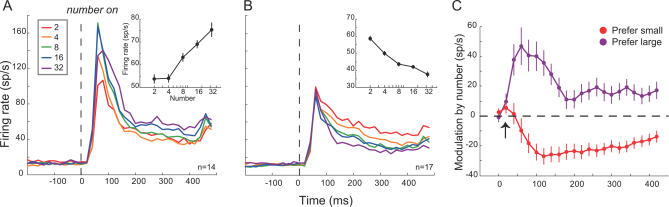
Numerical Selectivity Evolves Rapidly in LIP (A) For 14 neurons preferring large numerosity, averages were calculated in 20-ms bins for trials grouped by stimulus numerosity. Responses are aligned to stimulus onset (dashed vertical line at zero) and shown for the full stimulus period (400 ms). Numerosity is indicated by line color. Inset: average response (mean ± SE) during the epoch 50–450 ms following stimulus onset. (B) Average response of 17 neurons preferring small numerosity. Same conventions as (A). (C) Modulation of response for the two groups of neurons shown in (A) and (B). Linear regression was used to estimate the change in firing rate over the range of numerosities presented in a 40-ms window centered at the time point, sampled every 20 ms. Positive modulation indicates responses that increase with number; negative modulation indicates responses that decrease with increasing number. Arrow: time at which modulation first significantly differs from zero; subsequent time points are also significantly modulated by number (*p* < 0.05).

The time course of the neural response to visual arrays revealed two phases of discrimination. In the 200 ms before stimulus onset, baseline activity was low, at 14.2 ± 0.2 sp/s (Figure 3). Following a transient response that began approximately 40 ms after stimulus onset, we observed persistent modulations in activity related to the number of elements in the visual arrays (Figure 7). Figure 7A shows the average firing rate over the period of stimulus presentation for the 14 neurons that preferred larger numerosities. The transient increase in firing rate was higher for larger numerosities, and was followed by activity that decreased more quickly and to a lower extent with smaller numerosities. The level of activity that persisted throughout the rest of the interval remained above baseline and increased 22.4 sp/s over the range of numerical values presented (Figure 7A, inset; CI: 17.5 to 27.3, *F* = 80.22, *p* < 0.0001; Materials and Methods, Equation 2). Neurons that preferred small numerosities had a similar, but weaker, transient increase in activity that was larger for large numerosity arrays (Figure 7B). However, following the initial, brief positive modulation by numerosity, activity decreased more quickly and to lower levels with greater numbers of elements. Sustained activity in these neurons decreased 20.3 sp/s over the range of numerosities shown (Figure 7B, inset; CI: −23.1 to −17.5, *F* = 205.9, *p* < 0.0001; Materials and Methods, Equation 2), but overall, remained greater than baseline activity.

Both groups of neurons showed a larger initial neural response for larger numerical arrays before quickly diverging. For each group, modulation of activity by the number of stimulus elements was computed in 40-ms windows, calculated every 20 ms (Figure 7C). Initially, all neurons showed a reliable positive response modulation, indicating a larger response for larger numerosities. After approximately 40–60 ms, the responses for the two groups of neurons began to separate. Neurons that preferred large numerosities continued to show a positive modulation of activity (purple line) throughout stimulus presentation. However, the modulation of neurons that preferred small numerosities reversed such that larger numerosities evoked smaller responses (red). These two response profiles persisted throughout the entire stimulus viewing period.

**Figure 8:**
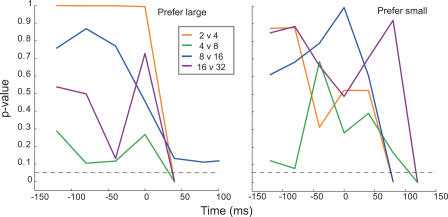
LIP Neurons Differentiate Numerosities Simultaneously For the two groups of neurons, “prefer large” and “prefer small,” trials were sorted by number, and the average response was calculated in 40-ms bins. At each time point, responses were compared for pairs of numerosities closest to each other using a one-tailed, two-sample *t*-test to determine the time at which the responses began to differ. The resulting *p*-value is plotted as a function of time. The time to first discrimination did not occur later for larger numerosities, suggesting that numerosity is encoded via a parallel rather than serial process.

Differential neuronal responses to numerosity emerged at a similar time across all values. Figure 8 shows the time at which the neural response to each numerosity began to differ from that of the value(s) closest to it (i.e., 2 versus 4, 4 versus 8, 8 versus 16, and 16 versus 32 [one-tailed, two-sample *t*-tests]). Before stimulus presentation (at time = 0), the neural responses for each pair of numerosities did not differ statistically from each other. For neurons that preferred large numerosities, the difference in firing rate for each pair of numerosities, except 8 versus 16, became significant approximately 40 ms after stimulus onset. The differences in firing rate emerged simultaneously for neurons that preferred small numerosities as well, although slightly delayed from the neurons that preferred large numerosities. For these neurons, the time to first discrimination did not occur at exactly the same time, but there were no systematic differences to suggest that discrimination of larger values occurred later. These results support parallel encoding of numerosity, with additional time required to generate negative modulations in response to increasing numerosity.

**Figure 9:**
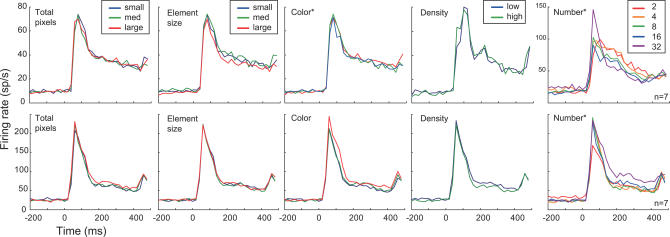
Numerical Sensitivity in LIP Does Not Reflect Low-Level Stimulus Attributes For 14 of the 31 number-sensitive neurons, stimuli that balanced total number of pixels, individual element size, color, or array density were all interleaved throughout each experiment. The top row shows responses for seven neurons that preferred small numerosities, and the bottom row shows responses for seven neurons that preferred large numerosities. In each panel, trials are divided according to one stimulus attribute only. Firing rate was calculated and then plotted in 20-ms bins.

The modulations in firing rate observed were not due to differences in other stimulus attributes. Because total number of pixels in the stimulus, size of the individual elements, and stimulus density typically covary with numerosity, several sets of stimuli were used that balanced these attributes while varying numerosity. In 17 of the 31 neurons significantly modulated by numerosity, these controls were instituted in a blockwise fashion. In the remaining 14 neurons, stimuli from all of the different types of controls were interleaved during recording. Neural responses on trials grouped by total pixels, element size, color, density, or numerosity are shown in Figure 9 for the 14 neurons tested with interleaved stimulus controls. We estimated the amount of modulation due to different stimulus attributes (Materials and Methods, Equation 4a). Seven of the neurons tested with interleaved stimulus controls preferred small numerosities (Figure 9, top row; modulation = −12.1 sp/s, CI: −16.4 to −7.8, *F* = 30.7, *p* < 0.0001, Equation 4a). The responses of these neurons during stimulus viewing were not affected by cumulative number of pixels (modulation = 0.003 sp/s, CI: −0.006 to 0.011, *F* = 0.32 *p* = 0.57, Equation 4a), element size (modulation = 0.002 sp/s, CI: −0.0002 to 0.005, *F* = 3.20, *p* = 0.08, Equation 4a), or density (modulation = 4.08, CI: −3.76 to 11.9, *F* = 1.05, *p* = 0.31, Equation 4a). For these neurons, there was modulation due to color, with responses 6.12 sp/s and 8.96 sp/s higher across numerosities for the red and green arrays, respectively, compared with blue arrays (CI_red_: 2.56 to 9.68, CI_green_: 5.39 to 12.53, *F* = 34.61, *p* < 0.0001, Equation 4a). When neural response was modeled with color and numerosity as factors, we found that significant modulation by numerosity remained (modulation by number = −9.70, CI: −13.2 to −6.2, nested *F* = 14.83, *p* < 0.0001; Materials and Methods, Equation 4b). The remaining seven of the neurons with interleaved stimulus controls showed greater activity for large numerosities (Figure 9, bottom: modulation = 24.0 sp/s, CI = 16.3 to 31.7, *F* = 37.4, *p* < 0.0001). In these neurons, we found no significant effect of total number of pixels (modulation = 0.002 sp/s, CI: −0.006 to 0.01, *F* = 0.17, *p* = 0.68, Equation 4a), element size (modulation = 1.34 sp/s, CI: −1.10 to 3.78, *F* = 1.16, *p* = 0.28, Equation 4a), or density (modulation = 2.88 sp/s, CI: −2.97 to 8.73, *F* = 0.93, *p* = 0.33, Equation 4a) on neural responses during stimulus viewing. There was a 15.1 sp/s larger response for red stimuli than green or blue (CI: 9.08 to 21.28, *F* = 13.25, *p* < 0.0001, Equation 4a). Again, modeling the neural response with both color and numerosity as factors showed that a significant modulation by numerosity persisted (modulation by number = 19.81, CI: 13.46 to 26.16, nested *F* = 18.7, *p* < 0.0001, Equation 4b). We performed an additional regression to test whether the effect of numerosity on neural response persisted when all other stimulus attributes were included. We found that including numerosity as a regression factor with cumulative number of pixels, element size, density, and color resulted in a significant improvement in the fit to the data (prefer small: modulation by number = −11.57 sp/s, CI: −20.60 to −2.54, nested *F* = 6.31, *p* = 0.01; prefer large: modulation by number = 17.93 sp/s, CI: 4.07 to 31.8, nested *F* = 6.43, *p* = 0.01; Materials and Methods, Equation 5). Just as neuronal responses were typically not affected by low-level stimulus attributes, neuronal activity also did not depend on whether a particular numerosity served as standard or deviant (prefer large: modulation = −2.47 sp/s, CI: −7.48 to 2.54, nested *F* = 0.94, *p* = 0.33; prefer small: modulation = −1.57 sp/s, CI: −4.51 to 1.37, nested *F* = 1.10, *p* = 0.29; Materials and Methods, Equation 3), thus effectively ruling out salience, attention, or reward expectations as factors that could account for the effect of numerosity on neuronal responses.

## Discussion

We found that a population of neurons in LIP encodes the number of elements in a visual array in a roughly monotonic manner. Parietal cortex has been implicated as a principal node of the neural circuit necessary for numerical processing, and previous evidence in monkeys has revealed neurons in VIP that are tuned to the cardinal value of elements within an array. Moreover, recent neuroimaging studies of responses to dot arrays like those used here have reported activation in human parietal cortex modulated by the number of elements in the array [10]. Accumulation of quantity has been proposed as a critical initial stage of enumeration, a process that could be served by “integrator” neurons in LIP [30,39]. Endorsing this idea, we found that a majority (>50%) of LIP neurons had activity that either increased or decreased with number. This response pattern differs both quantitatively and qualitatively from previous physiological studies [11–13,40].

All of the neurons that were modulated by numerosity showed an initial, transient response that increased with number (Figure 7). The transient increase in activity for larger arrays suggests an initial salience signal modulated by the number of possible objects to attend [41]. This transient response was followed by a longer-lasting signal correlated with accumulated numerical value. Rapid modulation by number, occurring in the first 40–60 ms, supports the hypothesis that numerical accumulation occurs simultaneously, in a parallel manner [12]. The initial, positive modulation is followed by persistent number-related activity that is analogous to temporal integration previously shown in LIP. Responses of LIP neurons while monkeys perform random-dot motion discrimination have been shown to increase or decrease proportionally to stimulus strength as a function of time [23,29], a process likened to temporal integration of visual motion information [28,30]. Our data suggest that, in addition to integrating with respect to time, LIP neurons also integrate the number of objects within their response fields.

The decrease in activity related to element number for neurons preferring small numerosities is also consistent with the hypothesis that some LIP neurons may signal oculomotor intentions. Multiple-element arrays could be interpreted as multiple saccade targets, so that the intention to look at any one would be divided among the *n* possibilities. Consistent with this idea, single neurons in superior colliculus show reduced responses with increasing number of potential saccade targets throughout the visual field [42] and suppression by multiple stimuli located within the same RF [43]. In light of these findings, it is worth noting that the monkeys in our study never made erroneous saccades to the numerical array.

Although lesion and neuroimaging studies in humans suggest that parietal cortex is critical for estimating numerical quantity, previous neurophysiological studies in monkeys have not shown an extensive representation of number in PPC. Nieder and Miller [12] found that approximately 20% of PPC neurons, but only 13% (10/77) of neurons in LIP, were modulated by the number of visual elements shown during the sample period in a numerical match-to-sample task. Further, those neurons responding to number did not resemble accumulators, but rather were tuned for particular cardinal values. Neurons in VIP were also found to have cardinal tuning for numerical stimuli presented sequentially (22%) as well as simultaneously (12%) [13]. Even when stimuli were presented in succession, number-related activity was tuned for a preferred position within the sequence, rather than changing systematically with number (see also [40]). VIP neurons typically have multimodal response fields aligned to the face and central visual field [44–46]; consequently, in the match-to-sample task used by Nieder and colleagues [12], numerical stimuli, ranging from one to five elements, were uniformly placed near central fixation. In contrast with neurons in VIP, LIP neurons typically have peripheral response fields located in the contralateral visual field [17–19]. Therefore, we tailored task geometry to the spatial sensitivity of the neural population tested by mapping the area of the RF that extended beyond the borders of the numerical cue. This may account for the higher proportion of number-sensitive neurons found here (>50%) than in previous studies. Most importantly, neurons in LIP represented numerical magnitude in a roughly monotonic manner, consistent with neural accumulation.

Neural network models of numerical representation [32,33] derive cardinal number from elements with just the type of graded coding shown by LIP neurons in this study. Although each model of numerical processing (Figure 1) includes an accumulator stage, they make different predictions about the manner in which numerical magnitude is represented. The accumulator in the mode-control model of Meck and Church [31] utilizes a serial mechanism for counting that produces an accumulated value linearly proportional to the number of events enumerated. The summation units in the neural network model of Dehaene and Changeux [32] use a parallel process to encode number as proportional to the total amount of activity following a normalization stage. Our findings are consistent with the summation units in the Dehaene and Changeux model. We observed a differential response to number that emerged simultaneously (Figure 8), regardless of whether the stimulus number was small or large, consistent with parallel encoding of numerosity. The Dehaene and Changeux model predicts that number is ultimately represented on a logarithmic scale by “numerosity” units, which are driven by the activation of summation units that exceed a particular threshold. Such numerosity units are consistent with neurons in VIP and PFC that were previously found to encode cardinal numerical value on a logarithmic scale. Our data do not strongly support either linear or logarithmic scaling of neuronal sensitivity to number in LIP. The graded responses we observed are also consistent with the neural network model proposed by Verguts and Fias [33], which predicts numerical summation represented by activity that increases or decreases monotonically with increasing quantity.

Our task was designed not only to consider spatial selectivity of LIP neurons, but also to reduce the influence of task demands such as reward expectation and training. Expectations about impending rewards have been shown to influence neural activity in such areas as LIP, PFC, and posterior cingulate cortex [24,35,36,47]. Such reward-modulation has been observed when the target of the upcoming reward was located in the neuron's RF (but see [35]). Here, we have positioned the target in the opposite direction of the RF. It is conceivable that the cue response would be influenced by reward magnitude or the frequency of a particular value's presentation, both of which differed according to a number's status as standard or deviant [9,24]. However, we did not observe this type of modulation. Training has also been shown to modulate neural responses. Monkeys performing the match-to-sample task based on numerosity had extensive training to explicitly categorize stimuli according to number [11]. This type of training has been shown to influence neural responses in PFC [48] and LIP [49]. Although our monkeys had ongoing experience with the task and their RTs suggests they at least implicitly processed the numerical array, there was no explicit requirement to discriminate number. This suggests the possibility that quantity may be encoded spontaneously in LIP, a hypothesis warranting further study in naive monkeys.

The neuronal sensitivity to number we observed is consistent with a spatial representation of accumulated magnitude. Coding of quantity within spatially selective response fields may offer a mechanism for the process of enumeration [33]. That there is a relationship between the representations of space and number offers potential explanation for the overlap of spatial and numerical deficits that characterize Gerstmann syndrome [50] which follows parietal damage, as well as the psychophysical observations of interactions between spatial and numerical judgments [14] (see also [15]). By taking the spatial selectivity of LIP neurons into account, we have found encoding of numerical magnitude in the activity of single neurons. The neurons sampled by Nieder and colleagues in other regions of PPC and PFC [11–13] may compute cardinal numerical representations based on inputs from neurons such as those reported here. In other words, these two classes of number-selective neurons may be the physiological instantiation of the summation units (ordinal) and numerosity units (cardinal) proposed in neural network models of numerical representation [32,33]. Quantity may thus be another aspect of visual information that is processed hierarchically within the dorsal visual stream, in which information such as color and orientation, typically associated with the ventral visual stream, is not lost at higher levels of processing, but is carried forward as more complex RFs are built [51,52].

## Materials and Methods

### Subjects.

Two adult rhesus macaque monkeys *(Macaca mulatta)* weighing 7.5–8.5 kg served as subjects. All procedures were approved by the Duke University Institutional Animal Care and Use Committee and were designed and conducted in compliance with the Public Health Service's Guide for the Care and Use of Animals.

### Surgical and training procedures.

A head restraint prosthesis and scleral search coil [53] were implanted during an initial, sterile surgical procedure performed under isoflurane inhalant anesthesia using standard techniques described in detail elsewhere [34]. Animals received postoperative analgesics for 3 d and antibiotic prophylaxis for 10 d after all surgeries. Following a 6-wk recovery period, animals were habituated to head restraint and trained to perform oculomotor tasks for fluid reward using custom software (http://www.ryklinsoftware.com). Horizontal and vertical eye positions were sampled at 500 Hz (Riverbend Instruments, http://www.riverbendinst.com) and recorded by computer. Visual stimuli were presented on a computer monitor (21 in, 1,024 by 768 pixels, 60 Hz refresh) 46 cm in front of the monkey. Once monkeys could perform the behavioral tasks, a second sterile surgical procedure was performed to place a stainless steel chamber (Crist Instruments, http://www.cristinstrument.com) over a 15-mm craniotomy over LIP (5 mm posterior and 12 mm lateral of stereotaxic 0,0; left hemisphere for monkey O and right for monkey W). Microelectrode recording began 1 wk following the recovery period.

### Microelectrode recording procedures.

Before each recording session, the cylinder was opened under aseptic conditions and repeatedly flushed with sterile saline. A Teflon grid (Crist Instruments) was secured in the cylinder and an X-Y micropositioner (Crist Instruments) and hydraulic microdrive (David Kopf Instruments, http://www.kopfinstruments.com) were mounted onto the cylinder. A tungsten steel (0.8–1.2 MΩ) electrode (FHC, http://www.fh-co.com) was drawn into a 23-gauge hypodermic tube, which was used to puncture the dura. Electrophysiological signals were amplified and filtered to exclude power-line noise and signals of the magnetic fields used to monitor eye position (band-pass, ∼0.2 to 5 kHz). Individual action potentials were identified in hardware by time and amplitude criteria, and the times of spike occurrences were recorded by computer.

### Neuron selection and spatial mapping of response fields.

A hallmark of LIP neurons is their spatial selectivity [17–19,54,55], so monkeys first performed a block of standard delayed-saccade trials to map each neuron's spatial RF. On each trial, a target was displayed in the periphery while the monkey fixated on a central point. Following a delay (700 to 1,500 ms), the fixation point disappeared and the monkey was rewarded with fluid for shifting gaze to the target. Spatial selectivity of each neuron was assessed online by initially presenting targets widely dispersed throughout the entire visual field. When elevated activity in one region was observed, saccade targets were placed at multiple locations within an approximately 12° × 12° area on approximately 80% of trials to test for an expanded area of elevated neural response. The target was located in the opposite hemifield on the remaining 20% of trials for comparison. Typically, 50–100 trials were used to map the RF in order to maximize data collection in the numerical task. Recording sites were localized to LIP using digital ultrasound imaging (monkey O, [34]) and histology (monkey W).

### Implicit numerical discrimination task.

On each trial, monkeys maintained fixation on a central point while a saccade target was placed in a random location either to the right (monkey O) or left (monkey W) of fixation (Figure 1A). Following a variable prestimulus delay, an array of 2, 4, 8, 16, or 32 circles was presented on the opposite side of the screen for 400 ms. When the fixation point was extinguished, the monkey shifted his gaze to the saccade target for a fluid reward. The likelihood that a particular numerosity was presented on each trial varied in a blockwise fashion. In each block, one number was selected as the *standard,* presented on 50% of trials. On each of the remaining trials, a *deviant* number was shown (Figure 1B). We encouraged monkeys to attend to the numerical stimulus by administering a standard reward (fluid delivered for 100-ms open solenoid time, for 0.10 ml) on successful standard trials and a larger reward (150-ms open solenoid time of fluid, for 0.15 ml) on all successful deviant trials. Once the RF was mapped, monkeys performed an average of 372 (range: 143–688) numerical discrimination trials. Each block consisted of approximately 100 trials, with two to five blocks per neuron.

### Stimulus controls.

Stimuli were generated using Matlab (The Mathworks, http://www.mathworks.com). Several sets of stimuli were constructed. All stimuli consisted of a set of 2, 4, 8, 16, or 32 circles plotted in random locations in a 7.0° × 7.0° square (135 × 135 pixels, except where noted). Circles were red, blue, or green on a black background. Two hundred stimuli were created for each number and each color, so that there were 3,000 bitmap images in each set of stimuli. Several sets of stimuli were used: (1) random size with balanced element density. Each element's radius was randomly varied between three to six pixels. Density was controlled by using a random walk to place circles in contiguous locations (each possible location a 15 × 15 pixel box); (2) balanced total pixels. The total number of pixels in each stimulus was chosen randomly from a uniform distribution of 402–2,513 pixels; (3) balanced element size. The radius for all the elements in a given stimulus was chosen from a uniform distribution of four to ten pixels; and (4) balanced element size and density. Element size was selected as in (2), but elements were placed within backgrounds of different sizes, ranging from 2.5° to 8.3° square.

### Analysis.

First, we tested whether saccade RT depended on the numerical distance between the standard and deviant value presented (Matlab; The Mathworks). For each trial, RT was measured as the time between fixation point offset and initiation of the eye movement. RT was modeled as:

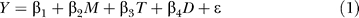
where *Y* is RT, *M* is monkey, *T* is the time delay between number cue offset and fixation point offset (0, 200, or 400 ms), and *D* is the absolute value of the difference between standard and delay for each trial (max = 30) and ɛ is random error. The coefficients, β_1_ through β_4_, were estimated using weighted least-squares regression. An *F* statistic was calculated using the principle of extra sum of squares [56]. This *F* statistic is referred to in the text as “nested *F,*” and its corresponding *p*-value is given, indicating the likelihood that the null hypothesis is true. Here, the null hypothesis, that β_4_ = 0, signifies that *D* had no effect on RT.

Neural data were processed offline to extract the time at which each trial event occurred as well as the time of each action potential. Neurons were first analyzed using ANOVA (Statistica; Statsoft, http://www.statsoft.com) to quantify whether there were any differences in firing rate related to quantity, without assuming a linear relationship (Figure 4 and Table 1). For each neuron, number and standard were included as factors to determine whether there were any differences in firing rate that depended on whether a particular numerical stimulus served as standard or deviant.

For each neuron, we tested whether neural responses were systematically related to numerosity as predicted using the model:

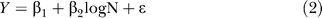
where *Y* is the spike rate in the period 50–450 ms after numerical cue onset, logN is the logarithmic transform of the numerosities 2–32, and ɛ is random error. The regression coefficients, β_1_ and β_2_, were estimated using weighted least squares. This model was also used to estimate modulation of neural response across the subpopulations of neurons preselected for large or small number preference (shown in Figure 7). *F* and *p*-values for the regression are reported in Results as well as β_2_ and its confidence intervals. Neurons were initially analyzed using both linear numerical value and the logarithmic transform of number. For neurons significantly modulated by number regardless of the scale used, we compared the *r*-values from each neuron's fit of response to linear number, and the logarithmic transform of number. To determine whether there was a statistically better fit of the data using a linear or logarithmic scale, we computed an *F* statistic, and corresponding *p*-value, from the ratio of the sum of squared deviances from the best fits of the average population data using both numerical scales (SS_lin_/SS_log_).

To determine whether firing rate depended on whether the numerical stimulus was presented as standard or deviant (Figure 6), we used the model:

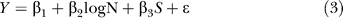
where *Y* is the spike rate in the period 50–450 ms after numerical cue onset, logN is the logarithmic transform of the numerosities 2–32, *S* is a dummy variable set to 1 on standard trials and 0 on deviant, and ɛ is random error. The null hypothesis that *S* did not affect the neural response (β_3_ = 0) was tested using nested regression as described in Equation 1.

To determine whether firing rate depended on stimulus attributes (Figure 9) that covary with numerosity we used the model:

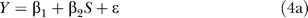
where *Y* is the spike rate in the period 50–450 ms after numerical cue onset, *S* is one of the variables that describe the particular stimulus presented on each trial, and ɛ is random error. Attributes tested were total pixels (cumulative number of pixels of the stimulus), element size (radius in pixels), and density (0 = low and 1 = high). Color was represented by two dummy variables, *I*_r_ (1 = red, 0 otherwise) and *I*_g_ (1 = green_,_ 0 otherwise). The fit yielded estimates of the modulation of firing rate due to the stimulus attribute modeled as well as a test of the null hypothesis that β_2_ = 0.

To determine whether modulation of neural responses due to number persisted in addition to modulation by other stimulus attributes, Equation 4a was modified to include number as a factor. We used the model:

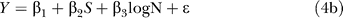
where *Y* is the firing rate in the epoch 50–450 ms after stimulus onset, *S* is an attribute listed above (Equation 4a), and logN is the logarithmic transform of the numerosities 2–32. The null hypothesis that numerosity did not affect the neural response when other stimulus attributes were included (β_3_ = 0) was tested using nested regression as described above.

Finally, to test whether the effect of number persisted when all of the stimulus attributes listed above were included, we modeled the neural response in the period 50–450 ms after stimulus onset *(Y)* as:

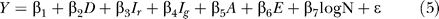
in which *D* is density (0 = low, 1 = high), *I*_r_ and *I*_g_ are dummy variables for color (as in Equation 4a), *A* is cumulative number of pixels, *E* is element size, and logN is the logarithmic transform of the numerosities 2–32. We used nested regression to test the null hypotheses that there was no significant effect of number on the neural response (β_7_ = 0) when the other stimulus attributes were known.

## Abbreviations

ANOVA: analysis of variance
CI: confidence interval
LIP: lateral intraparietal area
PFC: prefrontal cortex
PPC: posterior parietal cortex
RF: receptive field
RT: response time
sp/s: spikes/second
VIP: ventral intraparietal area
